# PrehospitaL Ultrasound in Undifferentiated DyspnEa (PreLUDE): a prospective, clinical, observational study

**DOI:** 10.1186/s13049-023-01070-4

**Published:** 2023-02-05

**Authors:** Elise Arem Gundersen, Peter Juhl-Olsen, Allan Bach, Martin Rostgaard-Knudsen, Bent Roni Ranghøj Nielsen, Søren Helbo Skaarup, Henrik Ømark Petersen, Jesper Fjølner, Morten Gustav Gerstrøm Poulsen, Morten Thingemann Bøtker

**Affiliations:** 1 Department for Research and Development, Prehospital Emergency Medical Services, Brendstrupgårdsvej 7, 2. th, 8200 Aarhus N, Central Denmark Region Denmark; 2 Department of Anesthesiology, Viborg Regional Hospital, 8800 Viborg, Denmark; 3 Department of Cardiothoracic and Vascular Surgery, Anesthesia Section, Aarhus University Hospital, 8200 Aarhus N, Denmark; 4 Department of Prehospital Emergency Medical Services, Central Denmark Region, Olof Palmes Allé 32, 1, 8200 Aarhus N, Denmark; 5 Department of Cardiology B, Aarhus University Hospital, 8200 Aarhus N, Denmark; 6 Department of Respiratory Medicine and Allergy, Aarhus University Hospital, 8200 Aarhus N, Denmark; 7 Department of Emergency Medicine, Horsens Regional Hospital, 8700 Horsens, Denmark; 8 Department of Anesthesiology, Randers Regional Hospital, 8930 Randers NØ, Denmark; 9 Emergency Medical Services, North Denmark Region, 9000 Aalborg, Denmark

**Keywords:** Dyspnea, Heart failure, Prehospital, Point-of-care ultrasound

## Abstract

**Background:**

Diagnostic uncertainty in patients with dyspnea is associated with worse outcomes. We hypothesized that prehospital point-of-care ultrasound (POCUS) can improve diagnostic accuracy.

**Methods:**

Prospective observational study of adult patients suffering dyspnea. Prehospital critical care physicians registered a suspected diagnosis based on clinical examination alone, performed POCUS of the heart and lungs, and finally registered suspected diagnoses based on their clinical examination supplemented with POCUS. Pre- and post-POCUS diagnoses were compared to endpoint committee adjudicated diagnoses. The primary outcome was improved sensitivity for diagnosing acute heart failure. Secondary outcomes included other diagnostic accuracy measures in relation to acute heart failure and other causes of dyspnea.

**Results:**

In total, 214 patients were included. The diagnosis of acute heart failure was suspected in 64/214 (30%) of patients before POCUS and 64/214 (30%) patients after POCUS, but POCUS led to reclassification in 53/214 (25%) patients. The endpoint committee adjudicated the diagnosis of acute heart failure in 87/214 (41%) patients. The sensitivity for the diagnosis of acute heart failure was 58% (95% CI 46%–69%) before POCUS compared to 65% (95% CI 53%–75%) after POCUS (*p* = 0.12). ROC AUC for the diagnosis acute heart failure was 0.72 (95% CI 0.66–0.78) before POCUS compared to 0.79 (0.73–0.84) after POCUS (*p* < 0.001). ROC AUC for the diagnosis acute exacerbation (AE) of chronic obstructive pulmonary disease (COPD) or asthma was 0.87 (0.82–0.91) before POCUS and 0.93 (0.88–0.97) after POCUS (*p* < 0.001). A POCUS finding of any of severely reduced left ventricular function, bilateral B-lines or bilateral pleural effusion demonstrated the highest sensitivity for acute heart failure at 88% (95% CI 79%–94%), whereas the combination of all of these three findings yielded the highest specificity at 99% (95% CI 95%–100%).

**Conclusion:**

Supplementary prehospital POCUS leads to an improvement of diagnostic accuracy of both heart failure and AE-COPD/-asthma overall described by ROC AUC, but the increase in sensitivity for the diagnoses of acute heart failure did not reach statistical significance. Tailored use of POCUS findings optimizes diagnostic accuracy for rule-out and rule-in of acute heart failure.

*Trial registration*: Registered in Clinical Trials, 05.04.2019 (identifier: NCT03905460) https://clinicaltrials.gov/ct2/show/study/NCT03905460?term=NCT03905460&cond=Dyspnea&cntry=DK&draw=2&rank=1.

## Background

Dyspnea is a frequent symptom among prehospital patients, and compared to other symptoms, prehospital dyspnea is associated with a high mortality [1–3]. Lack of early diagnostic clarification leads to prolonged hospitalization and increased mortality [4]. The etiology of dyspnea varies widely, and overlapping comorbidities challenge the diagnostic process among patients presenting with dyspnea in the emergency setting [5–9]. Importantly, the differentiation between pulmonary and cardiac causes of dyspnea is crucial, as treatments vary considerably. The use of point-of-care ultrasound (POCUS) in patients with respiratory symptoms improves diagnostic accuracy compared to clinical evaluation alone and identifies otherwise neglected critical illness in the emergency room [10, 11]. A recent prehospital pilot study demonstrated that a simplified ultrasound scan of the lungs examining only for B-lines is effective for ruling out acute heart failure but is not specific for rule-in [12]. Another small-scale prehospital study indicated that the presence of pleural effusion has a high sensitivity and specificity for acute heart failure [13]. Combining these findings with rough estimation of left ventricular (LV) systolic function into a more comprehensive examination may further improve diagnostic assessment. Thus, the objective of this study was to compare the diagnostic accuracy of physician-based clinical examination and clinical examination supplemented with POCUS in prehospital patients with dyspnea. B-lines, pleural effusion, and LV function was assessed for diagnosing acute congestive heart failure and the diagnostic value of other findings in relation to common causes of dyspnea were described. We hypothesized that supplementing the clinical examination with POCUS would increase the sensitivity of diagnosing acute heart failure compared to clinical examination alone.

## Methods

### Study design and setting

This was a prospective observational study reported according to the STARD guidelines and registered in Clinical Trials prior to conduction (identifier: NCT03905460). According to the Danish Act on Research Ethics Review of Health Research Projects, this quality improvement study did not require an approval from the research ethics committee system (Inquiry No. 208 / 2018). It was carried out in the Central Denmark Region, which is one of five political regions in Denmark, with a population of 1.3 million equivalent to 23% of the Danish population [14]. Ambulance services in the region are dispatched in two tiers. The first tier consists of ambulances staffed with emergency medical technicians and/or paramedics. The second tier consists of prehospital critical care teams staffed by physicians (anesthesiologists) dispatched either in one of ten regional rapid response vehicles and/or one of four nationally operated helicopter emergency medical services. Ultrasound competences among this group of physicians are heterogeneous, but the majority have extensive ultrasound experience from various courses and from in-hospital clinical use. POCUS competences were systematically implemented in prehospital critical care teams in our region from 2012 to 2018 by a combination of e-learning and through three hands-on courses held during continuous training courses. The use of POCUS was at physician discretion and in total, 40 of 136 physicians operating on one or more of the ten regional rapid response vehicles agreed to include patients for this study. These physicians routinely use POCUS for diagnostics in patients with dyspnea in the prehospital setting and agreed to report their clinical findings and the results of their ultrasound examinations.

### Participants

Patients attended by one of these 40 critical care team physicians in the Central Denmark Region between May 21, 2019 and December 31, 2021 were included. Inclusion criteria comprised age ≥ 18 years old and dyspnea as the primary complaint and respiratory rate > 25 min-1 and/or saturation < 95% and/or need for oxygen therapy based on a clinical judgement. Exclusion criteria were trauma prior to dyspnea and prior enrollment in the study. Drop-out criteria were POCUS not completed or patient not admitted to hospital following examination, as this precluded adjudication.

### Test methods

**Fig. 1:**
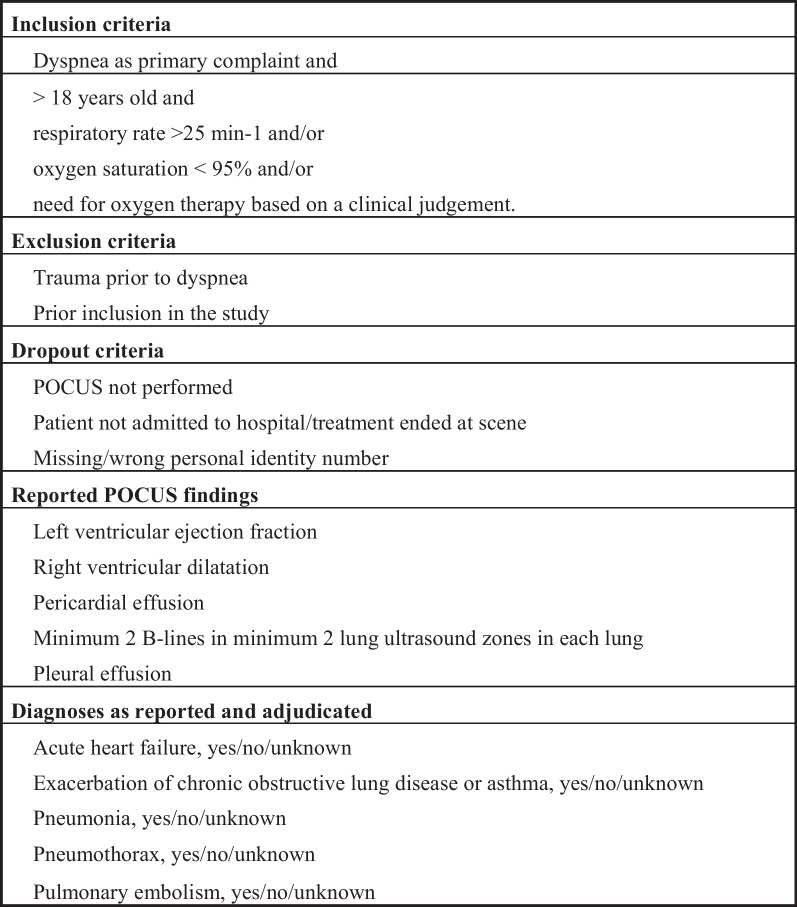
Study design—overview of inclusion, exclusion and dropout criteria in addition to POCUS findings reported and diagnoses reported and adjudicated

Patients were screened on a tablet on-scene and, following inclusion, the physician reported the suspected diagnosis according to Fig. 1 based only on patient history and physical examination. To include the possibility of high degree of diagnostic uncertainty, possible answers to each suspected diagnosis was Yes/No/Unknown. Subsequently, the physician performed a POCUS examination of the patient’s heart and lungs, with views and sequence of own preference, and registered predefined ultrasonographic findings as listed in Fig. 1. After completing the POCUS examination, the physician registered findings of the examination and a revised suspected diagnosis including supplementary POCUS findings (index test). Finally, the physician registered if findings of the POCUS examination changed prehospital treatment and/or triage. All POCUS examinations were performed with a SonoSite (Bothell, Washington, USA) iViz portable ultrasound scanner.

For patients in whom the pre- and/or post-POCUS diagnosis was registered as “unknown” by the physician, the patients were described, but excluded from diagnostic accuracy analyses.

An endpoint committee, blinded to the result of the POCUS examination, adjudicated the final diagnosis (reference standard) according to Fig. 1 based on electronic patient records (MidtEPJ, Systematics, Aarhus, Denmark) including results from laboratory analyses, and descriptions of x-ray, computed tomography scans and echocardiography, when performed. The committee consisted of three consultants in emergency medicine, cardiology and pulmonary medicine, respectively. Again, possible answers to the diagnosis were Yes/No/Unknown. The final diagnosis was determined by a 2:1 majority (Yes, No or Unknown) in the committee and this diagnosis was used to determine the accuracy of the prehospital pre- and post-POCUS diagnosis. An endpoint committee meeting was completed for consensus agreement on those patients where adjudicators answered three different things. Lack of consensus led to the answer "Unknown". Cases with "unknown" reference standard were described but excluded from diagnostic accuracy analyses.

### Data collection and outcome measures

All data including inclusion forms and patients journal material was registered in REDCap© electronic data capture tools hosted at Aarhus University [15, 16]. Baseline characteristics in terms of preexisting cardiopulmonary conditions, prescribed drugs and smoking habits at the time of admission, and results of examinations during admission were collected from electronic patient records.

The primary outcome was the change in sensitivity for diagnosing acute heart failure by adding POCUS of the hearts and lungs compared to clinical examination alone.

Secondary outcomes were (1) other diagnostic accuracy measures for acute heart failure with/without POCUS (receiver operating curve (ROC) area under the curve (AUC), specificity, positive predictive value (PPV), negative predictive value (NPV), positive and negative likelihood ratio (LR + , LR-)) (2) diagnostic accuracy measures of other etiologies of dyspnea (AE-COPD or AE-asthma, pneumonia, pulmonary embolism, pneumothorax) with/without POCUS, (3) diagnostic accuracy of specific POCUS findings (4) change of patient treatment due to POCUS findings and (5) change of triage due to POCUS findings.

Based on previous findings, we assumed a sensitivity of prehospital clinical examination for the diagnosis of acute heart failure of 59% and a prevalence of acute heart failure of 20% [17]). We assumed a possibility of increasing the sensitivity to a clinically significant level of 80% with POCUS. In a two-sided analysis with a proportion of discordant pairs of 30%, 255 ultrasound examinations were needed to achieve a strength of 80% and α 0.05. On September 24, 2020, we calculated the prevalence of patients with acute heart failure in our population to be 32% based on endpoint-committee adjudications on the first 100 patients and revised the sample-size calculation to 182 ultrasound examinations.

A total of 214 patients were included in this study, even though our revised sample-size calculation estimated a need of 182 POCUS examinations. The number included was higher than the sample size to ensure sufficient patient data with definite diagnostics at the end of inclusion. This was based on the observation of differences in the endpoint committee’s judgement of diagnoses early in the inclusion process.

Change in sensitivity and specificity for the diagnosis acute heart failure with supplementary POCUS was examined using McNemars exact test. Change in ROC AUC for the diagnosis acute heart failure was examined in paired analysis as proposed by Delong ER et al. [18]. No other comparative statistics were predefined, and the remaining data are presented descriptively. All statistical analyses were done in STATA MP version 17.0 (LCC StataCorp, Texas, USA). *P*-values < 0.05 were considered statistically significant. Data is presented as numbers and proportions with the corresponding 95% confidence interval (CI) where appropriate.

## Results

**Fig. 2:**
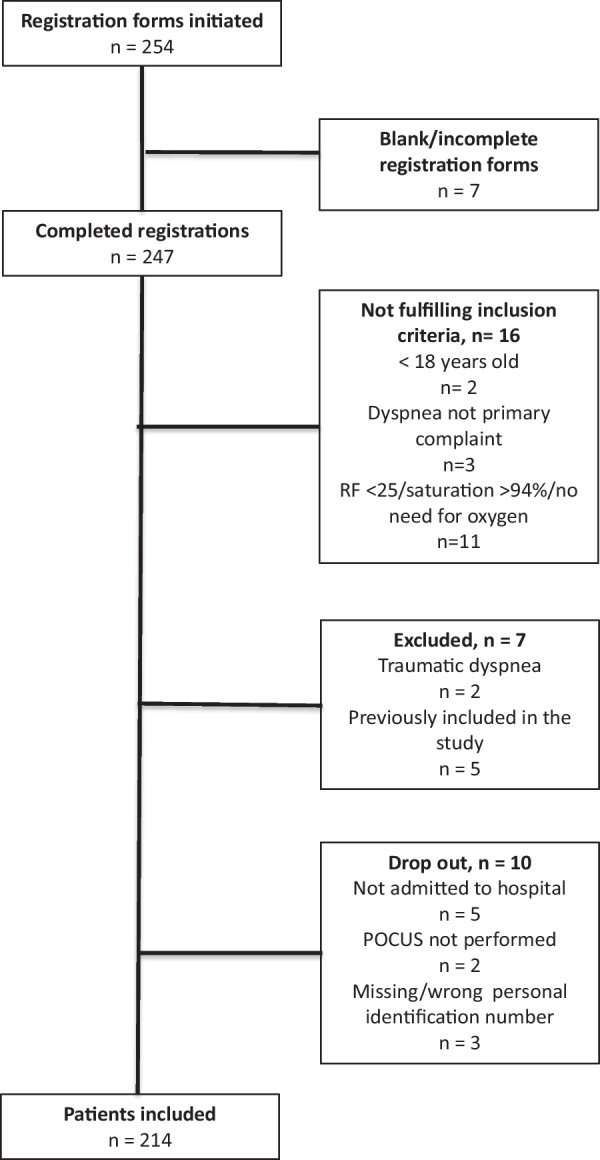
Inclusion flow chart

In total, 254 patients were screened for inclusion during the study period and of these, 214 patients were included (Fig. 2). Baseline characteristics of patients included in the study are presented in Table 1.

**Table 1:** Baseline characteristics for patients with dyspnea included (*n* = 214)

Characteristic	
Age, median (IQR)	76 (67.4–84.5)
Male, *n*(%)	115 (53.4)
*Pre-existing disease, n(%)*	
Ischemic heart disease	59 (27.6)
Heart failure	40 (18.7)
Other heart disease	94 (44.0)
Chronic obstructive pulmonary disorder / asthma	95 (44.4)
Restrictive lung disorder	4 (1.9)
Other pulmonary disorder	19 (8.9)
Chronic renal failure	33 (15.4)
*Prescribed medication, n(%)*	
ACE-inhibitor/ATII-antagonist	85 (39.7)
Beta blocker	77 (36.0)
Diuretics	100 (46.7)
Inhalation medication	91 (42.5)
*Smoking status, n(%)*	
Active smoker	54 (25.2)
Previous smoker	114 (53.3)

The numbers of patients with prehospital suspected diagnoses based on clinical examination alone and after supplementary POCUS are crossed with the results of the endpoint committee adjudication in Table 2.

**Table 2:** 2 × 2 tables with suspected diagnoses before and after POCUS crossed against endpoint adjudicated diagnosis

		PrePOCUS diagnosis		
		Yes	No	Total
*Acute heart failure*				
Endpoint committee diagnosis	Yes	46	34	80
	No	14	91	105
	Total	60	125	185
*AE-COPD/AE-asthma*				
Endpoint committee diagnosis	Yes	60	4	64
	No	28	110	138
	Total	88	114	202
*Pneumonia*				
Endpoint committee diagnosis	Yes	23	17	40
	No	30	102	132
	Total	53	119	172
*Pulmonary embolism*				
Endpoint committee diagnosis	Yes	2	3	5
	No	13	149	162
	Total	15	152	167
*Pneumothorax*				
Endpoint committee diagnosis	Yes	1	1	2
	No	12	169	181
	Total	13	170	183

		PostPOCUS diagnosis		
		Yes	No	Total
*Acute heart failure*				
Endpoint committee diagnosis	Yes	52	28	80
	No	9	105	114
	Total	61	133	194
*AE-COPD/AE-asthma*				
Endpoint committee diagnosis	Yes	56	6	62
	No	7	131	138
	Total	63	137	200
*Pneumonia*				
Endpoint committee diagnosis	Yes	22	22	44
	No	22	104	126
	Total	44	126	170
*Pulmonary embolism*				
Endpoint committee diagnosis	Yes	1	3	4
	No	4	179	183
	Total	5	182	187
*Pneumothorax*				
Endpoint committee diagnosis	Yes	1	1	2
	No	1	207	208
	Total	2	208	210

The most frequent prehospital suspected diagnosis was acute exacerbation of COPD or asthma in 95/214 (44%) of patients before POCUS and 69/214 (32%) after POCUS. The diagnosis of acute heart failure was suspected in 64/214 (30%) of patients before POCUS and 64/214 (30%) patients after POCUS. Diagnostic uncertainty regarding the acute heart failure diagnosis was present in 22/214 (10%) of cases before POCUS and in 13/214 (6%) after POCUS. The physician reclassified the presumptive diagnosis of acute heart failure following POCUS in 53/214 (25%) patients. The use of POCUS changed the prehospital treatment in 64/214 (30%) of patients and triage in 22/213 (10%) of patients.

### Diagnostic accuracy of presumptive diagnoses before and after POCUS

Diagnostic accuracy measures for the presumed diagnoses based on clinical examination alone (before POCUS) and after POCUS are presented in Table 3. The sensitivity for the diagnosis acute heart failure was 58% (95% CI 46%–69%) with clinical examination alone, compared to 65% (95% CI 54%–75%) after POCUS (*p* = 0.12). ROC AUC for the diagnosis acute heart failure was 0.72 (95% CI 0.66–0.78) before POCUS compared to 0.79 (0.73–0.84) after POCUS (*p* < 0.001). ROC AUC for the diagnosis AE-COPD/AE-asthma was 0.87 (0.82–0.91) before POCUS and 0.93 (0.88–0.97) after POCUS (*p* < 0.001).

**Table 3:** Diagnostic accuracy measures with 95% confidence intervals of presumptive diagnoses before and after POCUS

	ROC AUC	Sens	Spec	PPV	NPV	LR +	LR-
*Before POCUS*							
Acute heart failure (valid* *n* = 185)	0.72 (0.66–0.78)	57.5% (46–68)	86.7% (79–93)	76.7% (64–87)	72.8% (64–80)	4.31 (2.56–7.27)	0.49 (0.38–0.64)
AE-COPD/AE-asthma (valid* *n* = 202)	0.87 (0.82–0.91)	93.8% (85–98)	80.3% (72.6–86.6)	68.2% (57–78)	96.5% (91–99)	4.62 (3.3–6.47)	0.08 (0.03–0.2)
Pneumonia (valid* *n* = 172)	0.67 (0.59–0.76)	57.5% (41–73)	77.3% (69–84)	43.4% (30–58)	85.7% (78–92)	2.53 (1.68–3.82)	0.55 (0.38–0.8)
Pulmonary embolism (valid* *n* = 167)	0.66 (0.42–0.90)	40.0% (5–85)	92% (87–96)	13.3% (2–41)	98.0% (94–100)	4.98 (1.51–16.44)	0.65 (0.32–1.34)
Pneumothorax (valid* *n* = 183)	0.72 (0.23–1.0)	50.0% (1–99)	93.4% (89–97)	7.7% (0–36)	99.4% (97–100)	7.54 (1.7–33.46)	0.54(0.13–2.14)
*After POCUS*							
Acute heart failure (valid* *n* = 194)	0.79 (0.73–0.84)	65.0% (54–75)	92.1% (86–96)	85.2% (74–93)	78.9% (71–86)	8.23 (4.31–15.73)	0.38 (0.28–0.51)
AE-COPD/AE-asthma (valid* *n* = 200)	0.93 (0.88–0.97)	90.3% (80–96)	94.9% (90–98)	88.9% (78–95)	95.6% (91–98)	17.81 (8.61–36.82)	0.1 (0.05–0.22)
Pneumonia (valid* *n* = 170)	0.66 (0.58–0.74)	50% (35–65)	82.5% (75–89)	50% (35–65)	82.5% (75–89)	2.86 (1.77–4.63)	0.61 (0.45–0.82)
Pulmonary embolism (valid* *n* = 187)	0.61 (0.37–0.86)	25.0% (1–81)	97.8% (95–99)	20.0% (1–72)	98.4% (95–100)	11.44 (1.62–80.76)	0.77 (0.44–1.35)
Pneumothorax (valid* *n* = 210)	0.75 (0.26–1.0)	50.0% (1–99)	99.5% (97–100)	50% (1–99)	99.5% (97–100)	104 (9.47–1142.53)	0.50 (0.13–2.01)

*valid *n* is the number of patients with presumptive diagnoses before/after POCUS registered as yes/no (not "unknown") and endpoint committee adjudicated to yes/no (not "unknown")

### Diagnostic accuracy of specific POCUS findings

Specific POCUS findings are presented in Table 4. Diagnostic accuracy measures for the diagnosis of acute heart failure based on specific POCUS findings alone and in combination are presented in Appendix 1. Reduced left ventricular function and bilateral B-lines displayed similar diagnostic accuracies with sensitivity for acute heart failure of 75% (95% CI 64%–84%) and 71% (95% CI 60%–81%), and specificity of 73% (95% CI 63%–82%) and 76% (95% CI 67%–84%), respectively. The specificity was higher with bilateral pleural effusion at 92% (95% CI 85%–97%), but with lower sensitivity at 26% (95% CI 16%–37%).

**Table 4:** POCUS findings in patients with dyspnea (*n* = 214)

*LV function*	
Normal	94 (43.9%)
Mildly reduced	49 (22.9%)
Severely reduced	40 (18.7%)
Not possible	21 (9.8%)
Not examined	10 (4.7%)
*Dilated right ventricle*	
Yes	20 (9.4%)
No	146 (68.2%)
Not possible	38 (17.8%)
Not examined	10 (4.7%)
*Pericardial effusion*	
Yes	7 (3.3%)
No	191 (89.3%)
Not possible	5 (2.3%)
Not examined	11 (5.1%)
*Multiple B-lines left*	
Yes	98 (45.8%)
No	100 (46.7%)
Not possible	9 (4.2%)
Not examined	6 (2.8%)
*Multiple B-lines right*	
Yes	94 (43.9%)
No	100 (46.7%)
Not possible	12 (5.6%)
Not examined	8 (3.7%)
*Pleural effusion left*	
Yes	39 (18.2%)
No	148 (79.2%)
Not possible	8 (3.7%)
Not examined	19 (8.9%)
*Pleural effusion right*	
Yes	37 (17.3%)
No	146 (68.2%)
Not possible	9 (4.2%)
Not examined	22 (10.3%)
*Signs of PTX left*	
Yes	0 (0%)
No	182 (85.1%)
Not possible	4 (1.9%)
Not examined	28 (13.08%)
*Signs of PTX right*	
Yes	2 (0.9%)
No	183 (84.6%)
Not possible	3 (1.4%)
Not examined	28 (13.1%)

The highest achievable ROC AUC's for acute heart failure was seen when regarding any of severely reduced left ventricular function, bilateral B-lines or bilateral pleural effusion as positive, yielding ROC AUC of 0.75 (95% CI 0.69–0.81), which also yielded the highest sensitivity of 88% (95% CI 79%–94%). The highest specificity at 99% (95% CI 95%–100%), was observed with a combination of all three (severely reduced LV function, bilateral B-lines and bilateral pleural effusion) in combination, but with low sensitivity at 10% (5%–19%).

## Discussion

In this prospective observational study, supplementary POCUS improved the overall diagnostic accuracy for acute heart failure and acute exacerbation of COPD or asthma compared to clinical examination alone in patients with dyspnea in the prehospital setting. Although the increase in sensitivity for acute heart failure was modest and did not reach statistical significance, POCUS almost doubled the positive likelihood ratio for the diagnosis of heart failure. In addition, positive and negative likelihood rates for other diagnoses were either significantly improved or unchanged.

Compared to clinical examination alone, randomized controlled trials have demonstrated that a comprehensive cardiopulmonary POCUS increases the proportions of correct presumptive diagnose and the amount of patients with appropriate treatment in emergency departments [11, 19]. However, at present, no randomized controlled trials on the use of POCUS have been performed in the prehospital setting. Two smaller observational studies have examined physician-based POCUS in patients with respiratory symptoms in the prehospital setting but did not compare POCUS to clinical examination [12, 13]. The study by Laursen et al. examined lung ultrasound only and demonstrated high sensitivity of 94% and specificity of 77% for the diagnosis of cardiogenic pulmonary edema [12]. These numbers are very different from the POCUS-supported diagnosis of acute heart failure in the present study with sensitivity of 65% and specificity of 92% and the sole finding of bilateral B-lines with sensitivity of 71% and specificity of 76%. These differences may be explained by a highly selected number of physicians (seven) performing POCUS in the Laursen study. In addition, the patient group in the Laursen study is smaller (*n* = 40).

The findings of this study support the results by Neesse et al. that pleural effusion is associated with acute heart failure [13], but we were unable to reproduce the finding that all patients with acute heart failure had pleural effusions. We did find pleural effusion (and especially bilateral effusion) highly specific for acute heart failure with specificity of 92%, but we also demonstrated that the sensitivity is low at 26%. Thus, this seems to be a specific "rule-in finding", whereas the lack of bilateral pleural effusion does not rule-out acute heart failure. Our finding that POCUS changed patient management in 29% of patients is similar to Neesse et al. who reported changes in management in 25% of the patients.

In the present study, the sensitivity for acute heart failure increased less than expected with supplementary POCUS, so only slightly more patients were diagnosed with acute heart failure. However, the concurrent increase in specificity for acute congestive heart failure and increase in ROC AUC for both acute heart failure and AE-COPD or AE-asthma demonstrate that POCUS allowed for better differentiation of the cause of dyspnea. This is important because symptoms and clinical presentation for these diseases are shared, these diseases often co-exist, treatment modalities are different, and giving the wrong treatment impacts mortality especially for patients with heart failure [5, 7, 8]. Thus, the findings of this study support routine implementation of cardiopulmonary POCUS for patients with dyspnea in the prehospital setting, but POCUS is relevant not only for patients presenting with dyspnea. A recent a study of prehospital POCUS in a broader group of patients presenting with dyspnea, trauma, and cardiac arrest demonstrated a good correlation with in-hospital findings and changes in patient management in as many as 50% of patients [20]. Future studies could address how to systematically implement the use of POCUS in prehospital emergency medical services, especially among the group of physicians who do not use POCUS already.

### Strengths and limitations

A strength of this study was the comparison of clinical examination performed by experienced prehospital critical care physicians to examination supplemented with POCUS performed by the same physicians. This resembles a real-life approach and improves generalizability. Another strength was the comparison of the index test (i.e., supplementary POCUS) to a reference of expert committee adjudicated endpoints. In addition, the study was designed as a paired comparison of clinical examination and supplementary POCUS on the individual level that is highly clinically relevant and would not have been possible if the study had been designed as a randomized controlled trial.

Forty of 136 prehospital physicians agreed to register their presumed diagnoses and POCUS findings for this study. Although they represented all 10 rapid response vehicle units, they agreed to participate based on their interest in POCUS. This is a limitation to the generalizability of the study.

Of 214 patients with dyspnea included in this study, 41% suffered from acute heart failure. This proportion is higher than found in a similar study from our setting showing a prevalence of acute heart failure of 20% [17]. In March 2020, after 9 months of inclusion to the project, the SARS-CoV-2-virus pandemic hit Denmark. Due to infection control and governmental guidelines, the critical care team physicians were asked to avoid all unnecessary physical contact with patients. Therefore, during especially the first pandemic wave, POCUS was only preformed if judged to be strictly necessary and of significance for patient treatment. This may have resulted in a skewed population of more seriously ill patients included in this study, but how this affects diagnostic accuracies remains speculative.

Patients classified as "unknown" either before POCUS or after POCUS were not included in the comparative analysis. The alternative would be to consider unknown as a negative finding, but this is not in line with a clinical approach. The number of patients classified with "unknown" diagnoses decreased after POCUS. This is an important finding and the exclusion of patients reclassified from "unknown" to either yes or no following POCUS may have led to an underestimation of the effect of POCUS. However, the alternative would have been to include "unknowns" as a negative finding, which would be false and probably overestimate the effect of POCUS.

## Conclusion

Supplementing the clinical examination with POCUS lead to an improvement of diagnostic accuracy of both heart failure and AE-COPD/AE-asthma overall, as described by ROC AUC, compared to clinical examination alone, but the increase in sensitivity for the diagnoses of acute heart failure did not reach statistical significance. Finding any of severely reduced left ventricular function, bilateral B-lines or bilateral pleural effusion yields high sensitivity for the diagnosis of acute heart failure, correspondingly the combination of these three findings together is highly specific for acute heart failure.

## Data Availability

The datasets used and/or analyzed during the current study are available from the corresponding author on reasonable request.

## Appendix

See Table

**Table 5:** Diagnostic accuracy measures with 95% confidence intervals of reduced left ventricular function (any), B-lines, pleural effusion alone and in different combinations

	ROC AUC	Sens	Spec	PPV	NPV	LR +	LR −
Any reduced LV function (mild or severe) (Valid* *n*= 176)	0.74 (0.67–0.8)	74.7% (64–84)	73.2% (63–82)	69.4% (59–79)	78% (68–86)	2.79 (1.96–3.97)	0.35 (0.23–0.51)
Severely reduced LV function (Valid* *n*= 176)	0.69 (0.64–0.75)	43% (32–55)	95.9% (90–99)	89.5% (75–97)	67.4% (59–75)	10.4 (3.87–28.16)	0.59 (0.49–0.72)
B lines any side (Valid* *n*= 190)	0.72 (0.65–0.78)	76.8% (66–85)	66.7% (57–75)	63.6% (53–73)	79.1% (69–87)	2.30 (1.72–3.09)	0.35 (0.23–0.53)
Bilateral B-lines (Valid* *n*= 186)	0.73 (0.67–0.8)	70.9% (60–81)	75.7% (67–84)	68.3% (57–78)	77.9% (69–85)	2.92 (2.03–4.19)	0.38 (0.27–0.55)
Pleural effusion any side (Valid* *n*= 177)	0.64 (0.57–0.71)	42.7% (31–55)	85.3% (77–92)	68.1% (53–81)	66.9% (58–75)	2.9 (1.70–4.96)	0.67 (0.54–0.83)
Bilateral pleural effusion (Valid* *n*= 178)	0.59 (0.53–0.65)	25.7% (16–37)	92.3% (85–97)	70.4% (40–86)	63.6% (55–71)	3.34 (1.54–7.21)	0.81 (0.7–0.93)
Severely reduced LV function and bilateral b-lines (Valid* *n*= 190)	0.65 (0.59–0.7)	31.2% (21–43)	98.2% (94–100)	92.3% (75–99)	67.7% (60–75)	17.61 (4.29–72.36)	0.7 (0.6–0.82)
Severely reduced LV function and bilateral pleural effusion (Valid* *n*= 191)	0.55 (0.52–0.59)	11.5% (5–21)	99.1% (95–100)	90% (56–100)	61.9% (54–69)	13.04 (1.69–100.85)	0.89 (0.82–0.97)
Bilateral B-lines and bilateral pleural effusion (Valid* *n*= 181)	0.57 (0.52–0.62)	16.7% (9–27)	97.2% (92–99)	80% (52–96)	63.6% (56–71)	6.06 (1.77–20.71)	0.86 (0.77–0.95)
Any of severely reduced LV function or bilateral b-lines or bilateral pleural effusion (Valid* *n*= 169)	0.75 (0.69–0.81)	87.8% (79–94)	62.1% (51–72)	68.6% (58.8–77.3)	84.4% (73–92)	2.31 (1.75–3.06)	0.2 (0.11–0.36)
Severely reduced LV function and bilateral b-lines and bilateral pleural effusion (Valid* *n*= 191)	0.55 (0.51–0.58)	10.4% (4–19)	99.1% (95–100)	88.9% (52–100)	62.1% (55–69)	11.84 (1.51–92.8)	0.9 (0.84–0.98)

*Valid *n* is number of patients with the respective findings registered as yes/no (not "not possible" or "not examined") and endpoint committee adjudicated to yes/no (not "unknown")

5.

## Abbreviations

POCUS: Point of care ultrasound
ROC: Receiver operating curve
AUC: Area under the curve
AE: Acute exacerbation
COPD: Chronic obstructive pulmonary disease
LV: Left ventricular
PPV: Positive predictive value
NPV: Negative predictive
LR +: Positive likelihood ratio
LR −: Negative likelihood ratio
CI: Confidence interval
Sens: Sensitivity
Spec: Specificity
